# Supplementary material for: A bioinformatic analysis of the inhibin-betaglycan-endoglin/CD105 network reveals prognostic value in multiple solid tumors
Source: PLoS One. 2021 Apr 5;16(4):e0249558. doi: 10.1371/journal.pone.0249558 (PMC8021191; doi:10.1371/journal.pone.0249558)
Supplement: Attachment — Submitted filename: response.docx [file pone.0249558.s007.docx]

**Response to Academic Editor and Reviewers:**

- *“Please ensure that your manuscript meets PLOS ONE's style requirements, including those for file naming.”*

We do apologize for the divergence of style. We have thoroughly revised the documents, following both guidelines that were sent. We have edited:

- Headings and subheadings;
- Caption locations;
- In-text references of figures;
- Supplementary material text format;
- Title page;
- Figure and Supplementary material file names.
- *“We note that the grant information you provided in the ‘Funding Information’ and ‘Financial Disclosure’ sections do not match.”*

We regret the typo in the grant number. The sources of funding received for the research submitted to the journal are listed below. This information includes the name of granting agencies, grant numbers which have also been corrected in the manuscript data ' Funding section'.

**National Cancer Institute**
Award Number: R01CA219495 | Recipient: Karthikeyan Mythreye, Ph.D

**National Research, Development and Innovation Office Hungary**
Award Number: 2018-2.1.17-TET-KR-00001 | Recipient: Balázs Győrffy

**National Research, Development and Innovation Office, Hungary** 
Award Number: 2018-1.3.1-VKE-2018-00032 | Recipient: Balázs Győrffy

- *“Please ensure that you refer to Figure 3 in your text as, if accepted, production will need this reference to link the reader to the figure.”*

Our in-text references to the Figures have now been corrected /changed to “Fig 2 ***and*** 3” according to PloS manuscript formatting guidelines.

**REVIEWER #1**

***MINOR POINTS***

- *“TGFBR3 or betaglycan.….Since TGFBR3 has predictive value in cancer patients, authors should include in their discussion the potential impact of TGFBR3 changes on other pathways in discussion and similarly for endoglin CD105.”*

We appreciate the Reviewer’s point as both endoglin and betaglycan serve as co-receptors for other signaling pathways of the TGFβ superfamily and Wnt and FGF. Given the wide range of ligands we have now as suggested by the reviewer discussed the rationale and potential implications on these pathways in the broad context of carcinogenesis. These can be found on lines 509-518 in the final manuscript ( clean version).

- *“In Table S5, few known SMAD2/3 target genes were in the list suggesting non SMAD mechanisms downstream of this axis. Authors should discuss this as well.”*

We thank these reviewers for this point allowing us to highlight the significance of non-SMAD pathways in cancer. We have now added this to our discussion on lines 546-553 ( clean version)

- *“Considering the broad scope of the journal and its readers, at few places, the text may be further simplified and sentences shortened.”*

Changes have been made where appropriate.

**REVIEWER #2**

- *“The only problem is the quality of the images. The current resolution is low, so it is difficult to read.”*

We regret this and have now resubmitted higher resolution, 300-600 dpi, LZW-uncompressed, TIFF images which should offer better quality.
